# Chronic Strongyloides stercoralis infection in Fijian migrants to the UK

**DOI:** 10.1099/jmm.0.001925

**Published:** 2024-11-12

**Authors:** William D. Nevin, Jake Melhuish, Jayne Jones, Lucas Cunningham, James Dodd, Romeo Toriro, Matthew Routledge, Luke Swithenbank, Thomas D. Troth, Stephen D. Woolley, Angela Fountain, Claire Hennessy, Simon A. Foster, Charlotte Hughes, Mark R. Riley, Simran Rai, Russell Stothard, Edward D. Nicol, Mark Dermont, Duncan Wilson, David Woods, Lucy Lamb, Matthew K. O'Shea, Nicholas J. Beeching, Thomas Fletcher

**Affiliations:** 1 Department of Clinical Sciences, Liverpool School of Tropical Medicine, Liverpool, UK; 2 Department of Infectious Diseases, Imperial College London, London, UK; 3 Academic Department of Military Medicine, Royal Centre for Defence Medicine, Birmingham, UK; 4 Headquarters Army Medical Services, Camberley, UK; 5 Clinical Diagnostic Parasitology Laboratory, Liverpool School of Tropical Medicine, Liverpool, UK; 6 Department of Tropical Disease Biology, Liverpool School of Tropical Medicine, Liverpool, UK; 7 254 Multi-Role Medical Regiment, Cambridge, UK; 8 Department of Medicine, University of Cambridge, Cambridge, UK; 9 Royal Air Force Medical Services, High Wycombe, UK; 10 Institute of Microbiology and Infection, University of Birmingham, Birmingham, UK; 11 Defence Primary Healthcare, Lichfield, UK; 12 Household Cavalry Regiment, Bulford, UK; 13 Army Medical Services Support Unit, Camberley, UK; 14 Department of Military Medicine, Royal Centre for Defence Medicine, Birmingham, UK; 15 School of Biomedical Engineering & Imaging Sciences, King’s College London, London, UK; 16 Defence Public Health Unit, Research and Clinical Innovation Directorate, Defence Medical Services, Lichfield, UK; 17 Headquarters Defence Medical Services Group, Defence Medical Directorate, ICT Building, Edgbaston, Birmingham, UK; 18 Research and Clinical Innovation, Royal Centre for Defence Medicine, Birmingham, UK; 19 Carnegie School of Sport, Leeds Beckett University, Leeds, UK; 20 Northumbria Healthcare NHS Foundation Trust, Newcastle upon Tyne, UK; 21 University College London, London, UK; 22 Royal Free London NHS Foundation Trust, London, UK; 23 Centre of Defence Pathology, Royal Centre for Defence Medicine, Queen Elizabeth Hospital Birmingham, Edgbaston, Birmingham, UK; 24 Institute of Immunology and Immunotherapy, College of Medical & Dental Sciences, University of Birmingham, Edgbaston, Birmingham, UK

**Keywords:** eosinophilia, Fiji, helminth, migrant, *Strongyloides*

## Abstract

**Introduction.***Strongyloides stercoralis*, the human threadworm, is a parasitic nematode with global distribution, estimated to infect over 600 million people. Chronic infection is often asymptomatic, but hyperinfection and dissemination syndromes can occur in the immunosuppressed with high case fatality rates. Whilst strongyloidiasis is endemic in Fiji, its prevalence in Fijian migrant groups in the UK is unknown.

**Gap Statement.** No previous studies have been conducted on the prevalence of *Strongyloides* and other gastrointestinal parasites (GIPs) in Fijian migrants to the UK.

**Aim.** We conducted a cross-sectional study of the prevalence of GIPs in a Fijian migrant population.

**Methodology.** Participants completed a questionnaire on residence, travel and clinical symptoms and were asked to provide a serum sample for *S. stercoralis* IgG ELISA, venous blood samples for eosinophil count and a faecal sample for charcoal culture, multiplex real-time PCR (rtPCR) and microscopy after formalin-ethyl acetate concentration. Sequencing was performed on pooled *Strongyloides* larvae for nuclear 18S rRNA hyper-variable regions (HVRs) I and IV.

**Results.** A total of 250 participants (94% male) with median (range) age 37 (20–51) years entered the study, 15 (1–24) years since leaving Fiji. *S. stercoralis* IgG ELISA was positive in 87/248 (35.1 %) and 14/74 (18.9 %) had a GIP detected in faeces. This included 7/74 (9.5 %) with *Strongyloides* and 5/74 (6.8 %) with hookworms. Dermatological symptoms were more common in those with *Strongyloides,* and eosinophilia (>0.5×10^9^ cells per litre) was present in 55.6% of those with positive *S. stercoralis* IgG. rtPCR was the most sensitive faecal diagnostic test for *Strongyloides* and hookworms in faeces. Sequences of nuclear 18S rRNA for HVRs I and IV confirmed the presence of *S. stercoralis*.

**Conclusion.** This first cross-sectional study in Fijian migrants found a high rate of chronic infection with GIPs, particularly *S. stercoralis*. Faecal microscopy was insensitive compared to charcoal culture, rtPCR or serology, demonstrating the importance of specialist parasitological tests when investigating people with a suspected chronic infection. Our study highlights an overlooked burden of strongyloidiasis in the UK and has implications for screening and treatment programmes in Fiji and for migrants from Fiji.

## Introduction

*Strongyloides stercoralis*, the human threadworm, is a parasitic nematode that is estimated to infect over 600 million people [1]. Until recently, the global burden of *Strongyloides* infection was underestimated, probably due to the dominant use of diagnostic tools, such as the Kato-Katz method, which have poor sensitivity [2]. Diagnosis remains a challenge, with no agreed ‘gold standard’ diagnostic test [3]. The growing attention on *Strongyloides* as a pathogen is now evidenced by its formal inclusion within the soil-transmitted helminth (STH) group, as part of the World Health Organization’s strategy for control of neglected tropical diseases [4].

*S. stercoralis* has an auto-infective nature to its life cycle and infection can last for decades. Many infected individuals are asymptomatic [5], but some may experience recurrent gastrointestinal, dermatological and respiratory symptoms. In those who are immunosuppressed, particularly by corticosteroid therapy, a life-threatening hyperinfection and dissemination syndrome [5] can occur with a case fatality of around 50% [6]. *Strongyloides* infection may therefore present a risk to those who have travelled from an endemic to a non-endemic area for many years after migration, and diagnostic awareness may not be high amongst clinicians.

Surveillance data on gastrointestinal parasites (GIPs) infection in migrants to the UK are limited; most studies are performed on patient populations who have presented to healthcare settings, often after a finding of eosinophilia [7,9]. Routine screening for GIPs has been offered to Nepalese Gurkhas, a migrant group within the UK Armed Forces, since 2012 [10]. A recent retrospective study of this group found GIPs in 20.5% and *Strongyloides* seropositivity in around 5% [11]. Fijian-born personnel represent the second largest group of migrants from a GIP-endemic nation in the UK Armed Forces. An estimated 1750 British military personnel had Fijian recorded as their nationality in 2021 [12].

There are a few formal surveillance data on the prevalence of *Strongyloides* in adults in Fiji or in the Pacific Islands in general, and data in any age group are extremely limited. Coincident with surveillance of STH, studies have focused on school-age children, and most are several decades old [13]. It is possible that the prevalence of STH in Fiji has been reduced by longitudinal lymphatic filariasis (LF) control programmes [14], although pockets of filariasis still persist [15,16] and might cause cross-reactivity in *S. stercoralis* serological tests. Data on Fijian migrants are also rare, although a recent retrospective study of patients with *Strongyloides* in Auckland, New Zealand, found that a high proportion came from Pacific islands including Fiji [17].

Following the incidental diagnoses of *S. stercoralis* infections in several Fijian-born UK Armed Forces personnel who had lived in the UK for several years [18], we postulated that there is an unrecognized burden of asymptomatic infection in the UK. We undertook the first cross-sectional study of GIP infection in a population of Fijian migrants to the UK, using a combination of eosinophil counts, serology and traditional and molecular faecal diagnostic techniques.

## Methodology

### Participant recruitment

We recruited Fijian-born personnel from throughout the UK Armed Forces by advertising through primary care centres, augmented by word of mouth through excellent social networks in this group. Study days were held at five locations throughout the UK for potential participants in 2021–2023. Participants were eligible if they were over the age of 18, were currently serving in the UK Armed Forces and had migrated to the UK from Fiji. Following the provision of written informed consent, participants were asked to fill in a brief questionnaire about prior residence and travel and relevant persistent symptoms, including diarrhoea, abdominal pain, blood in faeces, rashes, cough or shortness of breath and unexplained weight loss and fevers, and to provide venous blood samples and a faecal sample. For the research analysis, questionnaires and samples were anonymized with a participant number, linked to diagnostic samples for the same individual.

Data from the first blood and faecal tests are analysed in this report. All those with a positive/equivocal serological or faecal result or eosinophilia were invited for full clinical review and treatment as indicated, and limited diagnostic results following these visits are also discussed separately.

### Differential white cell count

Samples of venous blood in EDTA were transported at room temperature within 24 h to the clinical haematology laboratory at the Royal Liverpool University Hospital, where differential white blood cell counts were performed as per standard protocols. Eosinophilia was defined as a peripheral blood eosinophil count >0.5×10^9^ l^−1^ [19].

### *S. stercoralis* IgG ELISA

Clotted blood samples were transported at room temperature within 24 h and refrigerated on arrival at the Clinical Diagnostic Parasitology Laboratory of the Liverpool School of Tropical Medicine. An aliquot of serum was collected from each sample. IgG antibodies to *S. stercoralis* were detected using a commercial *S. stercoralis* IgG ELISA (DRG Instruments, Marburg, Germany) and processed as described by Nevin *et al*. [11]. The threshold of positivity was defined as an optical density (OD) of ≥0.200. High negative values (−) were defined as an OD value between 0.185 and 0.199. Weak positives (+) were defined as an OD value between 0.200 and 0.249. Standard positives were defined as an OD value between 0.250 and 0.999 (++). A strong positive (+++) was defined as an OD value ≥1.000. This approach was informed by comparison with a previously designed in-house IgG ELISA and validated using known positives, known negatives, known cross-reactive samples and samples used in a national laboratory evaluation scheme [11].

### Faecal analysis

Faecal samples were transported unrefrigerated without preservatives and were typically processed within 18–24 h of passage. Each sample was examined by light microscopy after formalin-ethyl acetate (FEA) concentration, and a faecal charcoal culture was performed as described by Nevin *et al*. [11].

### Multiplex real-time PCR

Faecal samples underwent DNA extraction and real-time PCR (rtPCR) using the method described by Cunningham *et al*. [20]. Faecal samples were screened using a two-tube rtPCR assay, with Reaction 1 screening for *Giardia duodenalis*, *Cryptosporidium* spp., *Entamoeba histolytica*, *Entamoeba dispar* and the phocine herpesvirus (PhHV-1) internal positive control. Reaction 2 screened for *Ascaris lumbricoides*, *Trichuris trichiura*, *Strongyloides* spp., *Schistosoma* spp. and hookworms (both *Ancylostoma duodenale* and *Necator americanus*). These two assays used pre-published primers and probes [21] and were carried out in 20 µl reactions on the Rotor-Gene platform (Qiagen, Manchester, UK). For a single 20 µl, 2 µl of DNA template and 10 µl of iQ Supermix (Bio-Rad, Watford, UK) were used. The thermocycler conditions were as follows: hold at 95 °C for 15 min and then 50 cycles of 94 °C for 15 s, followed by 60 °C for 60 s. A cycle threshold (*C*_t_) cut-off value of 38 was introduced and samples with a *C*_t_ of <38 were considered positive and those of ≥38 were considered equivocal. A negative test was defined as no amplification in the presence of an appropriate internal positive control reaction.

### Molecular analyses

A sample of pooled *Strongyloides* larvae was harvested from the charcoal cultures of three participants. Each sample was centrifuged at 5000 r.p.m. for 5 min and a 10 µl pellet was taken. DNA was extracted by adding 48 µl of Tris-EDTA (TE) buffer and 2 µl of proteinase K (20 mg µl^−1^) to each sample. This was vortexed for 10–20 s and then centrifuged for 10–20 s at 7000 r.p.m., followed by incubation for 60 min at 56 °C. Samples were then vortexed and centrifuged again prior to further incubation for 15 min at 93 °C.

Hyper-variable regions (HVRs) I and IV of the nuclear 18S rRNA gene were targeted for sequencing, using published primers and the thermal PCR cycle from Barratt *et al*. [22] Primers are shown in Table S1 (available in the online version of this article).

Amplified DNA was separated by gel electrophoresis and suitable amplicons with clear visible bands were excised. The excised bands were centrifuged at 8000 r.p.m. for 5 min through a high-density polyethylene filter to separate the liquid DNA product, and the assessment of DNA concentration was performed using NanoDrop ND-1000. The product was purified using ExoSAP-IT (Thermo Fisher, Waltham, MA, USA).

Sanger sequencing was performed by Source BioScience^TM^. Sequences were analysed and aligned using mega 11 software. Low-quality reads at the ends of chromatograms were trimmed and erroneous base calls were corrected manually. The nucleotide Basic Local Alignment Search Tool (blast) for nucleotides was used to find the most similar sequences from the National Center for Biotechnology Information non-curated core_nt reference database.

### Statistical analysis

Prior residence in Fiji was classified by respondents as predominantly urban, rural or both. Participants were also stratified by reported residence in a province with a medium human development index (HDI) or one with a high HDI, according to the Subnational Human Development Database [23]. Data were analysed using SPSS V28 and GraphPad Prism 10 software. Odds ratios (ORs) were calculated using single and multiple logistic regression analyses, with the exception of the OR for detection of GIPs in those with non-pathogenic protozoa and OR for symptoms in participants with positive faecal tests for *S. stercoralis*, both of which were calculated via direct comparison of odds due to the small group sizes. For comparisons between populations, either a *χ*^2^ test was used or a Fisher’s exact test if any of the groups had an *N* of <5. For comparison of continuous variables, the Mann–Whitney *U* test or the Kruskal–Wallis test was used as appropriate due to the non-normal distribution of data.

## Results

### Demography and symptoms

Out of 250 participants recruited, 235 (94.0 %) were male; the median (range) age was 37 (20–51) years. Age was not normally distributed (Fig. S1). The country of birth was available for 244 participants, of whom 242 (99.2 %) were born in Fiji. All participants had migrated to the UK between 1998 and 2021, with a median (range) 15 (1–24) years since migration (interquartile range 3–20). The majority (160/244, 65.6%) also had a history of travel to another *S. stercoralis* endemic country. Of the 224 participants who provided further details about their previous residence in Fiji, 129 (52.9 %) had primarily lived in an urban environment, 100 (41.0 %) in a rural environment and 15 (6.1 %) split their time equally between a rural and an urban environment. Full details of previous residence in Fiji and other travel destinations are provided in Tables S5 and S6.

One or more persistent symptoms were reported by 94/244 [38.5 %, 95 % confidence interval (CI) 32.6–44.8] participants. This included 52 (21.3 %) with gastrointestinal symptoms, 42 (17.2 %) with rashes, 33 (13.5 %) with cough or shortness of breath, 42 (17.2 %) with subjective fevers and 4 (1.6%) with weight loss (Table S7). Associations between symptoms and diagnostic results are presented later.

### Faecal results

Seventy-four participants provided a stool sample, of whom 14 (18.9 %) had at least one faecal test positive/equivocal for a GIP (Table 1). The most common pathogen was *S. stercoralis,* in seven cases (9.5%), followed by the hookworm spp. in five (6.8 %), *E. histolytica* and *E. dispar* co-infection in one (1.4%) and one equivocal rtPCR result for *G. duodenalis*. If rtPCR results above the clinical *C*_t_ cut-off value (≥38) are discounted, we identified GIP infection in eight participants (10.8%, 95 % CI 5.3–20.2); four *S*. *stercoralis* (5.4%), three hookworms (4.1%) and a single *E. histolytica* case. No multiple pathogen infections were found.

**Table 1.** Summary of positive/equivocal faecal tests in 14 study participants with GIPs

Organism	Participants with any positive/equivocal faecal result		rtPCR		FEA and microscopy	Charcoal culture
	No.	Prevalence,% (95 % CI)	Positive	Equivocal		
*S. stercoralis*	7	9.5 (4.4–18.5)	4	3	1	2
Hookworm	5	6.8 (2.6–15.2)	3	2	0	1
*E. histolytica*	1	1.3 (0.0–8.0)	1	0	1	
*G. duodenalis*	1	1.3 (0.0–8.0)	0	1	0	
Any	14	18.9 (11.5–29.4)	8	6	2	

CIConfidence intervalFEAFormalin-ethyl acetate

### S. stercoralis

All seven participants with a positive/equivocal faecal test for *S. stercoralis* had DNA detected on rtPCR (4/7 positive, 3/7 equivocal); 2/7 were positive on charcoal culture and 1/7 was positive on FEA microscopy. All had a positive *S. stercoralis* IgG ELISA (median OD 1.604) and eosinophilia (Table 2). As such, all those with an equivocal *S. stercoralis* rtPCR were considered as likely true positive results for the purpose of further analyses discussed later. The median eosinophil count of 1.4×10^9^ l^−1^ was significantly higher than that of participants with negative faecal results (median 0.2×10^9^ l^−1^, *P*<0.001). The median (range) age of the seven participants was 38 (24–42) years and the median (range) time since migration to the UK was 16 (1–21) years; 3/7 participants had not travelled to any other *S. stercoralis* endemic country.

**Table 2.** Diagnostic test results in seven participants with positive/equivocal faecal rtPCR for *S. Stercoralis* For definitions of semi-quantitative results for faecal PCR and serological results, see text.

Age (years)	Years since migration to UK	Eosinophil count (10^9^ l^−1^)	FEA microscopy	Charcoal culture	rtPCR	*S. stercoralis* IgG ELISA OD	*S. stercoralis*IgG ELISA result
38	16	0.7	+	+	+	1.037	+++
42	20	1.7	−	+	+	2.642	+++
29	1	2.5	−	−	+	0.626	++
24	2	3.0	−	−	+	0.351	++
25	3	1.3	−	−	Equivocal	1.929	+++
29	21	1.4	−	−	Equivocal	1.825	+++
41	19	0.7	−	−	Equivocal	1.604	++

### Other GIPs

Five individuals had a positive or equivocal faecal test for hookworm species. The median age of these five individuals was 29 years. Three had eosinophilia and the median (range) eosinophil count was 0.5 (0.1–4.0) ×10^9^ l^−1^, not significantly different from participants with negative faecal results. The longest time reported by a hookworm-positive participant since the last being in Fiji was 3 years. Details of those with a positive hookworm result can be found in Table S3.

One participant had infection with *E. histolytica* and one other had an equivocal PCR result for *G. duodenalis*. Protozoa that have disputed pathogenicity or are generally thought to be non-pathogenic were found in 6/74 (8.1 %) participants. The presence of non-pathogenic protozoa was not significantly associated with having a positive/equivocal faecal test for a pathogenic GIP (OR 0.85, 95 % CI 0.07–6.29, *P*=1.00).

### *S. stercoralis* IgG ELISA

The *S. stercoralis* IgG ELISA was positive in 87/248 (35.1 %, 95 % CI 29.4–41.2) participants (Table S2). There was no difference in the median ages of those with a positive IgG ELISA (39 years) and those with a negative IgG ELISA (36 years, *P*=0.108).

**Fig. 1.**
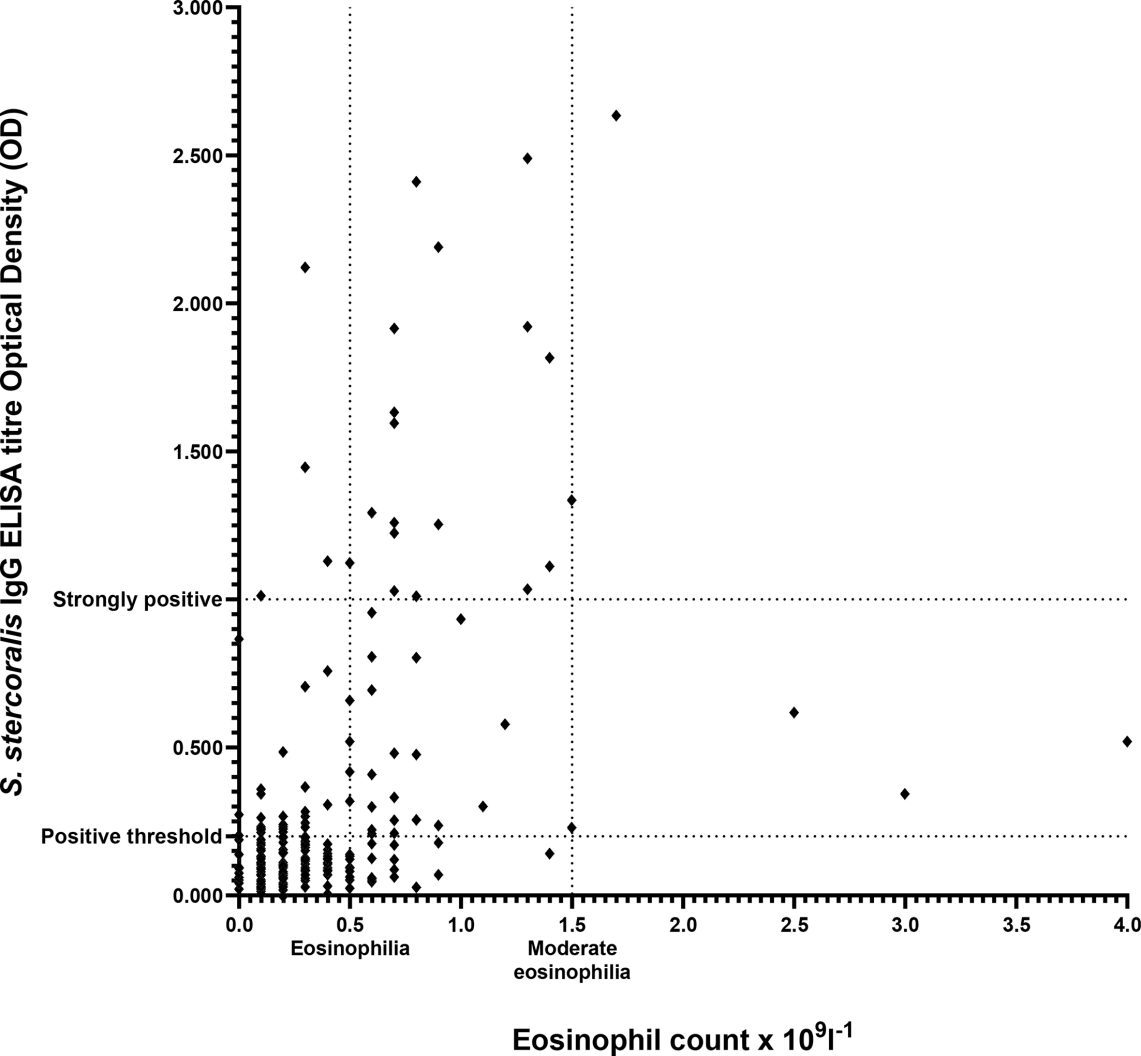
Scatter plot of *S. stercoralis* IgG ELISA OD versus eosinophil count. Thresholds of *S. stercoralis* positivity (OD≥0.200) and strong positivity (≥1.000) are shown on the *y*-axis. Thresholds of eosinophilia (>0.5×10^9^ l^−1^) and moderate eosinophilia (>1.5×10^9^ l^−1^) are shown on the *x*-axis.

Overall, 68/229 (29.7 %, 95 % CI 24.1–35.9) participants had eosinophilia, which was more common in those with a positive *S. stercoralis* IgG ELISA (45/81, 55.6%, 95 % CI 44.7–65.9) compared to 23/148 (15.5 %, 95 % CI 10.52–22.3) with a negative ELISA (*P*<0.001). The median eosinophil count was significantly higher in those with a standard positive (0.5×10^9^ l^−1^) or strongly positive (0.7×10^9^ l^−1^) *S. stercoralis* IgG ELISA compared to those with negative or weakly positive results (both 0.2×10^9^ l^−1^) (*P*<0.001, *P*=0.035, *P*<0.001 and *P*<0.001, respectively, Kruskal–Wallis test). There was no significant difference in median eosinophil counts between those that were IgG negative and those that were IgG weakly positive (*P*=1.00). There was a moderate positive correlation between increasing *S. stercoralis* IgG ELISA serological titre and increasing eosinophil count (Spearman’s rank-order correlation *r*_s_=0.461, *P*<0.001) (Fig. 1).

**Fig. 2.**
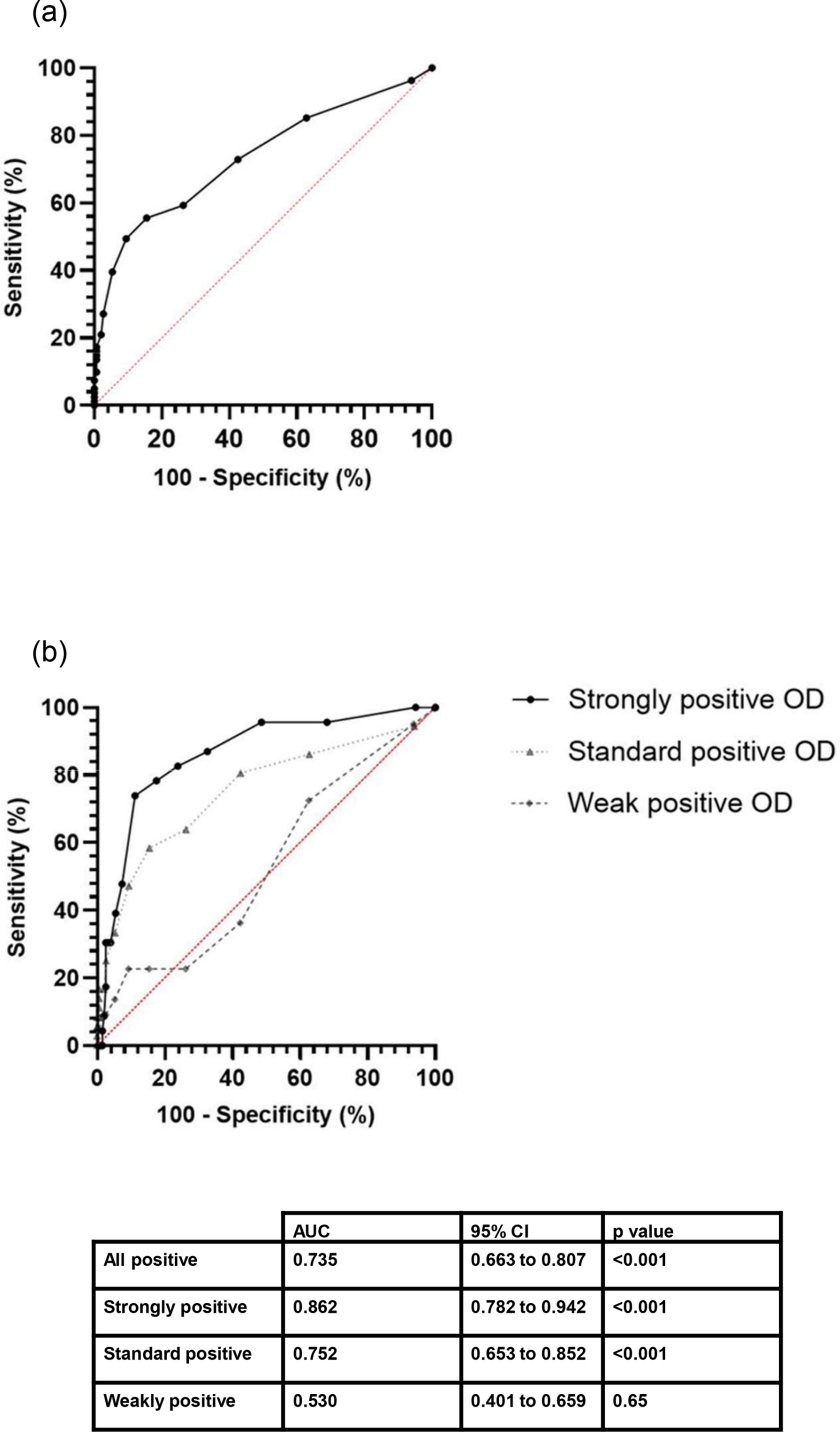
ROC curves for eosinophil count prediction for *S. stercoralis* IgG ELISA IgG positivity. All positive serological results are grouped together on graph A. Graph B stratifies serological results, by whether they were strongly positive, standard positive and weakly positive results (see text for definitions). AUC, area under the curve.

Receiver operating characteristic (ROC) curves were created to explore the sensitivity and specificity of eosinophil count as a diagnostic test to predict positivity of *S. stercoralis* IgG ELISA (Fig. 2]) if a diagnostic cut-off eosinophil count of 0.5×10^9^ l^−1^ was used, sensitivity was 55.6% (95 % CI 44.7–65.9) and specificity was 84.5% (95 % CI 77.8–89.4).

### Comparison of *Strongyloides* diagnostic methods

The sensitivity and specificity of each diagnostic method for *S. stercoralis* were compared against two reference standards: positive/equivocal rtPCR and positive *S. stercoralis* IgG ELISA. All those with an equivocal *S. stercoralis* rtPCR were considered as positive results for the purpose of this analysis (Table 3).

**Table 3.** Sensitivity and specificity of diagnostic tests versus (i) rtPCR and (ii) *S. stercoralis* IgG ELISA Values used for the calculation of sensitivity and specificity can be found in Table S4.

	Versus rtPCR			Versus IgG ELISA		
	Charcoal culture	FEA microscopy	IgG ELISA	Charcoal culture	FEA microscopy	rtPCR
Sensitivity, % (95 % CI)	28.6(3.7–71.0)	14.3(0.4–57.9)	100(59–100.0)	5.9(0.7–19.7)	2.9(0.07–15.3)	20.6(8.7–37.9)
Specificity, % (95 % CI)	100.0(94.6–100.0)	100.0(94.6–100.0)	58.5(45.6–70.6)	100.0(90.8–100.0)	100.0(90.8–100.0)	100.0(90.8–100.0)

ROC curves were created to explore the sensitivity and specificity of eosinophil count to predict positive/equivocal faecal tests for *S. stercoralis*. The area under the curve was 0.950 (95 % CI 0.897–1.000), suggesting that rising eosinophil count was a good predictor of faecal positivity for *S. stercoralis* (Fig. S3).

### Migration and travel history

Logistic regression was performed to examine possible associations between a positive *S. stercoralis* IgG ELISA and travel to an endemic area, living in a rural environment or living in a medium HDI province. When analysed individually, both living in a rural environment and living in a medium HDI province were associated with seropositivity, but only living in a rural area remained significant in the multivariate analysis (OR 2.04, *P*=0.02). Full results of logistic regression can be found in Table S8. Of the participants who provided a faecal sample, 16 had no travel history apart from Fiji and the UK, and of these 3/16 had *S. stercoralis*. Of the participants who provided a serological sample, 84 had never travelled to another endemic area, and 23/84(27.4 %) had a positive *S. stercoralis* IgG result.

### Symptomatology

One or more symptoms were reported by 94/244 (38.5 %, 95 % CI 32.6–44.8) participants overall, and by all 7 (100 %) with a positive/equivocal faecal test for *S. stercoralis,* compared to 27/65 (41.5 %) who did not (*P*=0.004). Of those with positive *S. stercoralis* IgG, 49/86 (57.0 %, 95 % CI 46.4–66.9) reported symptoms compared to 45/157 (28.7 %, 95 % CI 22.2–36.2) of those with negative serology. Although the presence of any symptoms, GI symptoms and rashes were all associated with positive * S. stercoralis* IgG ELISA seropositivity in univariate logistic regression analysis; the only significant association remaining after multivariate analysis was the presence of rashes (OR 3.44, *P*<0.01). Full results of logistic regression can be found in Table S9.

Two participants described classic larva currens rashes [24]. Both had strongly positive *S. stercoralis* IgG ELISA titres and eosinophilia. One was positive for *S. stercoralis* in all faecal tests and one had negative faecal examinations.

### Molecular identification of species

#### 18S rRNA HVR I

Each sample yielded identical 435 bp sequences. A nucleotide blast search most closely matched all three sequences with * S. stercoralis*, accession number LC537179.1 [25], with a 100% query cover and 100% identification. This sequence was obtained from *Strongyloides* taken from faecal samples of healthy volunteers in Myanmar. *Strongyloides stercoralis* sequences from a captive Sumatran orangutan (OM423625.1) [26] and environmental dog faeces (MK778085.1) in Australia [26] also had a 100% identification match (with 100 and 99% query cover, respectively). The most closely related genotype, according to the classification of Barratt *et al*. was II [22].

#### 18S rRNA HVR IV

Each sample gave identical 255 bp sequences. Nucleotide blast found a high number of matches with *S. stercoralis* sequences with 100% query cover and 100.00 % identification. The top match was with accession number MN498130.1, sourced from faecal samples of dogs in Thailand, followed by MN498129.1, sourced from faecal samples of human agricultural workers in Thailand [27], and MK468664.1, sourced from environmental dog faeces in Australia [28]. The sequences corresponded to genotype variant A [29].

## Discussion

We believe this to be the first cross-sectional study of GIP infection in Fijian migrants to any country, and it is the first study in any Fijian population to use a combination of traditional faecal diagnostics with molecular and serological investigations. We have demonstrated a high rate of infection, with 18.9% of participants having a positive or equivocal faecal test for a pathogenic GIP and 35.1% having a positive *S. stercoralis* IgG ELISA. These findings highlight the need for wider surveillance and treatment programmes for *Strongyloides* in Fiji and for diagnostic surveillance of strongyloidiasis in migrants to the UK.

There are limited data on the current prevalence of *S. stercoralis* within Fiji. A study of schoolchildren published in 2016 found a single positive case using the Baermann method. The same study examined 173 faecal samples using PCR, reporting a prevalence of 3.5% [30]. Whilst the authors of that paper did not describe a *C*_t_ cut-off, they used the same method described by Verweij *et al*., who included all values in their analyses [31]. Using the same primers, 7/74 (9.5 %) of our study population had positive/equivocal faecal results, including two with positive cultures and one positive on microscopy. Older reports demonstrated a higher prevalence of *Strongyloides*, with rates up to 50% depending on location [13,32, 33]. It is possible that rolling out mass drug administration (MDA) for LF from 2001 [34] may have reduced STH infection rates, as demonstrated in other countries [14].

Recent data for other STHs are also limited. Prevalence rates of about 10–20 % have been demonstrated using Kato-Katz and FEA microscopy [34,35], with older reports describing much higher rates of infection [32]. In contrast, we found a hookworm prevalence of 6.8%. One reason for the lower prevalence in our population may be that participants had no further exposure following migration to the UK and had cleared infections without treatment. Of those in our study population who had migrated to the UK within the last 10 years and provided a stool sample, 6/24 (25 %) had a positive/equivocal faecal result for STH (three each with hookworm or *S. stercoralis*). Our study should not, therefore, be taken as evidence of a low hookworm prevalence amongst adults in Fiji.

We found that reporting prior residence in a predominantly rural location was associated with having a positive *Strongyloides* serological test. However, STH infections in Fiji have been reported in both urban and rural areas [36,37].

Whilst we believe this is the first cross-sectional study on migrants from Fiji, Cutfield *et al*. performed a 10-year retrospective analysis of 691 *Strongyloides* patients in New Zealand, of whom 19% were born in Fiji. They described a similar rate of seropositivity (39%) to that found in our study. Whilst their work was on a retrospective patient cohort with a higher median age (63), it supports our findings of high rates of *S. stercoralis* in Fijian migrants. They also described a much higher rate of positive microscopy of 69/250 compared with 1/74 in our study. Their participants were a patient population, most of whom were immunosuppressed and so may have had higher faecal positivity rates [17]. Our positive microscopy rate (1.4%) was similar to that found in a global meta-analysis of *Strongyloides* infections in migrants (1.8%). However, we found a much higher seroprevalence (35.1%) versus the 17.3% previously reported in migrants from east Asia and the Pacific [38].

Previous studies in Fiji have largely relied on microscopic techniques, without access to culture or molecular methods. Microscopy has been reported to be insensitive for chronic *Strongyloides* [11,21, 39], consistent with our findings; only 1/7 case of *S. stercoralis* was found using FEA microscopy. The use of the Baermann technique or a culture method such as charcoal or agar can improve sensitivity [40,41], and we found further cases on charcoal culture compared with microscopy alone (2/7).

rtPCR was the most sensitive faecal test method and was also highly specific. For *Strongyloides*, all cases found via charcoal culture or microscopy were also detected by rtPCR. This was supported by findings at a clinical follow-up; faecal samples were provided by further 36 patients who had not yet given a sample. *S. stercoralis* was detected in 8/36 of them by PCR, of whom three had positive charcoal culture and one had positive FEA microscopy. Two hookworm positives were also found, both PCR positive, and one positive on charcoal culture and FEA. Whilst multiple different PCRs have been developed for *Strongyloides*, few have undergone validation [42]. However, the assay we used, developed by Verweij *et al.* [31], is one of the most widely utilized in both clinical and research settings [42]. Increased sensitivity using this assay compared with that using traditional faecal methods has been previously described[21,43]. However, a meta-analysis of molecular techniques reported that the sensitivity of PCR was only 56.5% when compared to *Strongyloides* serology or faecal methods [44].

Serological diagnosis has frequently been reported to have higher diagnostic sensitivity than other methods [11,45, 46]. It was the most common positive test in our cohort, with a sensitivity of 100% in patients with any positive faecal test, albeit in a small number of cases. Interpretation of the clinical relevance of seropositivity for *S. stercoralis* is difficult, especially in people with no symptoms. A positive result may represent resolved a past infection as sero-reversion may take months or even years following resolution [47]. Cross-reactions with other helminths, especially filariasis, are well documented [48]. LF caused by diurnally subperiodic *Wuchereria bancrofti* [49] was still highly endemic in Fiji in the early twenty-first century [50], and despite MDA remains endemic in some areas [15,16]. Norsyahida *et al*. analysed the sensitivity and specificity of a commercial assay in those with proven *Strongyloides* and found it to be 83.6 % when those with other helminths were included [51]. Ongoing investigations for potential serological cross-reactions in our cohort will be reported separately.

At least one symptom was present in 57% of those with positive serology and in all seven who had positive/equivocal faecal tests for *S. stercoralis*. Taking into consideration the wide CIs for the latter (59.6–100.0 %) due to small numbers, this is similar to the symptom rate described in other observational studies (62.6%) and a previous meta-analysis of 20 studies using faecal methods, which identified symptoms in 50.4% of cases [52].

As in the meta-analysis, we found that gastrointestinal and dermatological presentations were the most common symptoms in those with a positive test. The presence of rashes was the only symptom or symptom group significantly associated with a positive *S. stercoralis* IgG in multivariate logistic analyses in our study. Only two participants reported classic larva currens. Whilst supported by some literature [53], this contrasts with a previous study of chronic *S. stercoralis* infection in the UK that reported a 70% rate of larva currens [54]. However, the study population was much older (mean age 65 years) and many were admitted for intensive investigation, with increased opportunities to explore symptoms in detail.

We found no significant associations between serological positivity and respiratory symptoms, weight loss or subjective fevers in univariate or multivariate logistic regression models. Fevers are more common in *Strongyloides* hyperinfection, although they have been described in people who are immunocompetent, including a patient who had acquired *S. stercoralis* in Fiji [55,56].

Eosinophilia was present in all seven patients with positive faecal tests and 55.6% (45/81) of those with positive serology. Some authors, such as Naidu *et al*., have suggested that eosinophilia is a poor predictor for *Strongyloides* infection in migrants, finding a raised count in only 25% of their serologically positive cohort [57]. This contrasts with a study of migrants to Spain, where 95% of patients with positive serology had eosinophilia [58], and a large group of chronically infected former prisoners of war of whom 66% had eosinophilia [54]. The meta-analysis by Buonfrate *et al*. reported eosinophilia in 69.3% [52] of patients, and our results support the assertion that the majority of patients with *S. stercoralis* infection will have eosinophilia.

A rising eosinophil count was a good predictor that a patient would have a positive faecal result for *S. stercoralis*, as well as predictive of a positive *S. stercoralis* IgG ELISA. However, we found that a diagnostic cut-off eosinophil count of 0.5×10^9^ l^−1^ had a sensitivity of only 55.6%, with over 40% (36/81) of serologically positive participants having a normal eosinophil count. We also note that at the clinical follow-up, three individuals with a faecal positive test had eosinophil counts that fluctuated between normal and high levels. Fluctuations in eosinophil counts with strongyloidiasis have been described by previous authors [59,61]. Therefore, whilst an important clue, a normal eosinophil count does not exclude the diagnosis of strongyloidiasis.

The rtPCR used in this study has been found to be accurate to the genus level, with potential detection of *Strongyloides* species other than S. *stercoralis* [62]. Few studies on *Strongyloides* have used molecular methods to identify the infecting species and most have assumed it to be *S. stercoralis*. Different species have implications with regard to possible zoonotic transmission, clinical features and potential for autoinfection [42]. Other human infections have been reported in the western Pacific area, such as *Strongyloides fuelleborni kellyi* in Papua New Guinea [63] and *Strongyloides fuelleborni fuelleborni* in southeast Asia [64]. Using 18S rRNA HVR I and HVR IV, our samples matched most closely with *S. stercoralis* haplotype II A [22], which has been described in humans and dogs [28]. Whilst large numbers of stray dogs are present in Fiji, we are not aware of any recent studies that have identified *Strongyloides* in the canine population. Further studies on the potential for zoonotic transmission in Fiji are required.

Our study had several limitations. Although all serving Fijian personnel were eligible and invited to attend, it is possible that those with symptoms or concerns about *S. stercoralis* would have been more likely to take part, affecting the generalizability of our findings to all Fijian personnel. The study included predominantly adult males who had lived in the UK for a median of 15 years since leaving Fiji, and results cannot be extrapolated directly to the general population in Fiji. For our sensitivity and specificity calculations, the number of positives was low, and as a result, CIs were large. We recognize the low numbers of faecal samples provided by participants compared with serum and blood samples. Efforts were made to mitigate this, such as providing stool collection instructions and kits in advance and offering collection and postage of samples. However, these did not result in a large increase in samples. The reasons for the lack of sample provision may be complex and multifactorial and should be investigated to aid future studies in this population. Given the further cases of *S. stercoralis* found at the clinical follow-up, it is likely that additional stool sampling would have identified more faecal positive cases.

Details of prior residence, travel and persistent symptoms were all self-reported in an unsupervised questionnaire that had not been rigorously validated; answers were not verified further. This may have skewed the significance of any potential associations between travel and the prevalence of infection. All participants were serving Armed Forces personnel and many could have undertaken occupational activities in endemic areas that involved soil exposure and hence potential infection. However, individuals should have access to good quality footwear and clothing during military travel, potentially reducing infection risk. Furthermore, positivity rates for STH infections were similar even in those who had never been to any endemic area other than Fiji when compared to those in the whole group. The only significant residential risk factor for *Strongyloides* infection in multivariate analysis was rural residence in Fiji, and many study participants reported at the clinical follow-up that they were frequently barefoot whilst in Fiji. As infections with *S. stercoralis* may persist for decades, these could have been acquired in Fiji at any time.

This analysis focuses on the results of one set of blood and faecal test, which is recognized to be less likely to identify infection than more intensive investigation [11,65]. As in other studies, PCR was the most sensitive faecal test modality [21,43]. The detection of DNA alone may not necessarily indicate active infection, although PCR has been evaluated as having a high specificity for *S. stercoralis* in meta-analysis [44]. Possible causes of a false positive rtPCR result include laboratory contamination. However, our samples were often run with other clinical samples, and there was no increase in positivity rates to suggest this. Furthermore, the diagnostic laboratory undergoes regular quality assurance. The interpretation of equivocal rtPCR results may be difficult, but in this study, all participants with equivocal results had both eosinophilia and positive *S. stercoralis* serology, suggesting they had true infections. All these cases were offered clinical follow-up and treatment.

Our findings suggest that there is a proportion of the adult population in Fiji with undiagnosed chronic *S. stercoralis* infection. Further study is required to confirm this and to evaluate the impact of LF MDA campaigns since 2001 that used albendazole. In 2017, the World Health Organization recommended the addition of ivermectin in areas that have not met the epidemiological targets for elimination of LF [66]. This may have the additional benefit of reducing *S. stercoralis* prevalence in these areas. A single dose of 200 mg/kg ivermectin has a cure rate of around 86% against *S. stercoralis* [67], higher than that after multiple doses of albendazole [68].

In conclusion, our findings have demonstrated for the first time that chronic *S. stercoralis* infection is present in a number of Fijian migrants to the UK. Sequencing confirmed the presence of *S. stercoralis* strains, known to be prevalent in the Pacific region. Infection was more common in those with a history of rashes or with eosinophilia but was also present without either of these indicators. As in other studies, diagnostic tests confirmed greater sensitivity of rtPCR than faecal microscopy or charcoal culture to detect STH in single faecal samples from a migrant population. rtPCR has the advantage over culture methods of increased speed of processing and does not require as much contact with potentially infective material [69]. However, it remains a relatively expensive technique that requires significant resources, and further study is required on its utility over traditional methods in a low-resource environment.

These results suggest that some adult male migrants from Fiji have chronic infections with potential for severe complications later in life and some may have no indicator symptoms or peripheral eosinophilia. Whilst the UK Migrant Health guide states that up to 20% of migrants from endemic countries may have helminth infections on arrival to the UK (https://www.gov.uk/guidance/helminth-infections-migrant-health-guide) [70], we have demonstrated it to be higher in certain populations. Programmes should be designed to identify and screen Fijian migrants to the UK for STH and to treat those with positive results or to treat all such migrants presumptively [71,74]. The application and success of such policies should also be studied in women and children arriving from Fiji. Systematic evaluation of the prevalence and risk factors for *S. stercoralis* infection in Fiji should employ modern diagnostic techniques to inform the need for changes to mass screening and treatment programmes for STHs, especially *S. stercoralis*.

## supplementary material

10.1099/jmm.0.001925 Uncited Supplementary Material 1.

## Abbreviations

AUC: area under the curve
blast: Basic Local Alignment Search Tool
bp: base pair
CI: confidence interval
C_t_: cycle threshold
DNA: deoxyribonucleic acid
ELISA: enzyme-linked immunosorbent assay
FEA: formalin-ethyl acetate
GIP: gastrointestinal parasite
HDI: human development index
HVR: hyper-variable region
IgG: immunoglobulin G
LF: lymphatic filariasis
MDA: mass drug administration
OD: optical density
OR: odds ratio
PhHV-1: phocine herpesvirus
ROC: receiver operating characteristic
rRNA: ribosomal ribonucleic acid
rtPCR: real time polymerase chain reaction
STH: soil-transmitted helminth
TE: tris-EDTA
UK: United Kingdom
