# Supplementary material for: Chronic Strongyloides stercoralis infection in Fijian migrants to the UK
Source: J Med Microbiol. 2024 Nov 12;73(11):001925. doi: 10.1099/jmm.0.001925 (PMC11556407; doi:10.1099/jmm.0.001925)
Supplement: Uncited Supplementary Material 1. [file jmm-73-01925-s001.pdf]

Supplementary Material

Supplementary figure 1. Histograms of age (A) and years since migration to the UK (B).

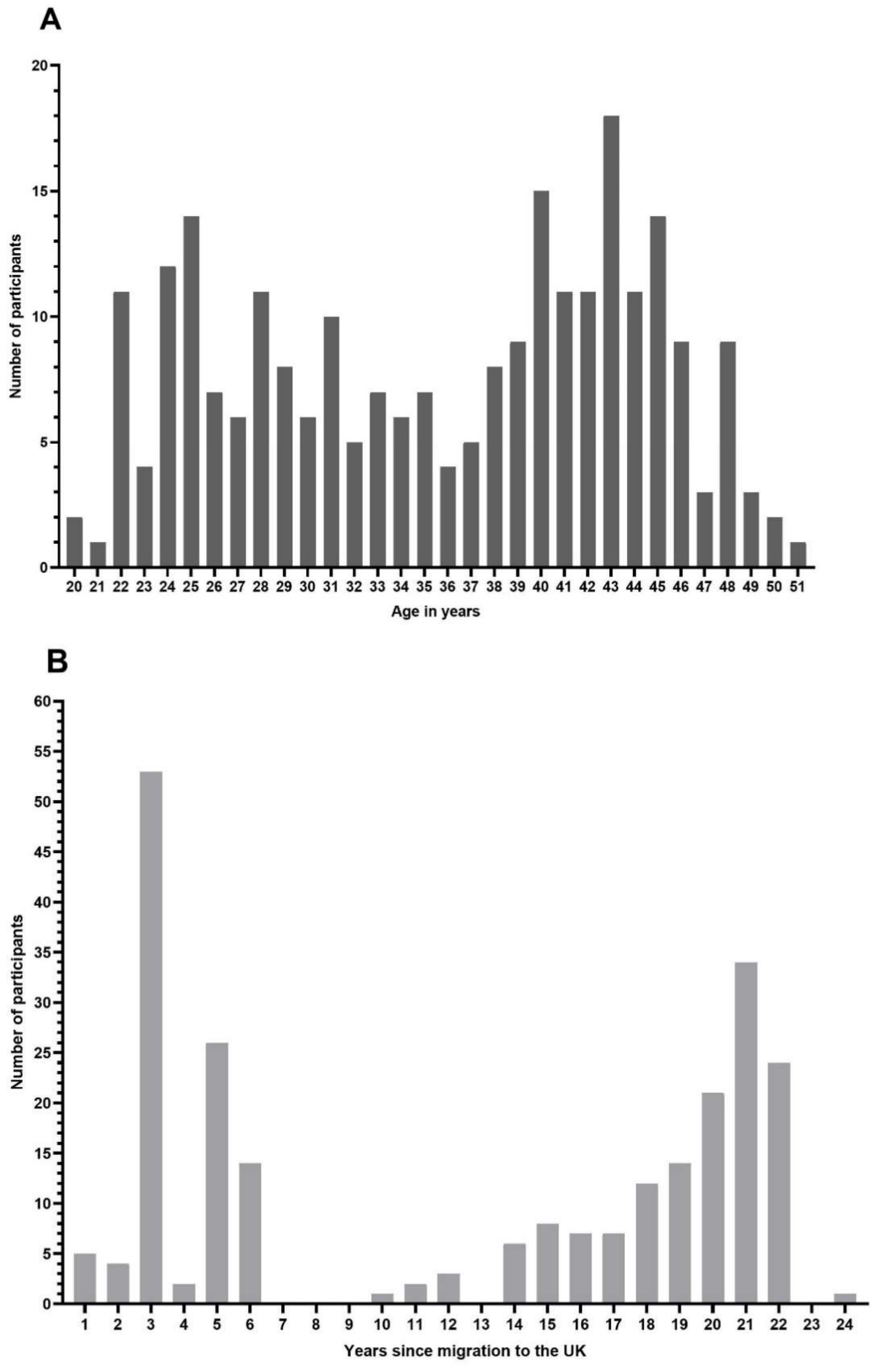

Supplementary figure 2. Map of participant *Strongyloides stercoralis* IgG ELISA seropositivity by primary area of residence in Fiji. Number of participant and percentage IgG positive are shown for each Division (Central, Eastern, Northern, Western) and for each province.

Western Division

N = 49

S. Stercoralis IgG ELISA seropositivity = 40.81%

Province	N	IgG ELISA +ve	% IgG ELISA positive
Ba	33	12	36.40
Ra	4	3	75.00
Nadroga-Navosa	12	1	41.70

Central Division *

N = 164

S. Stercoralis IgG ELISA seropositivity = 31.70%

Province	N	IgG ELISA +ve	% IgG ELISA positive
Rewa	101	28	27.72
Tailevu	33	15	45.50
Naitasiri	14	3	21.40
Serua	13	4	30.80
Namosi	3	2	66.70

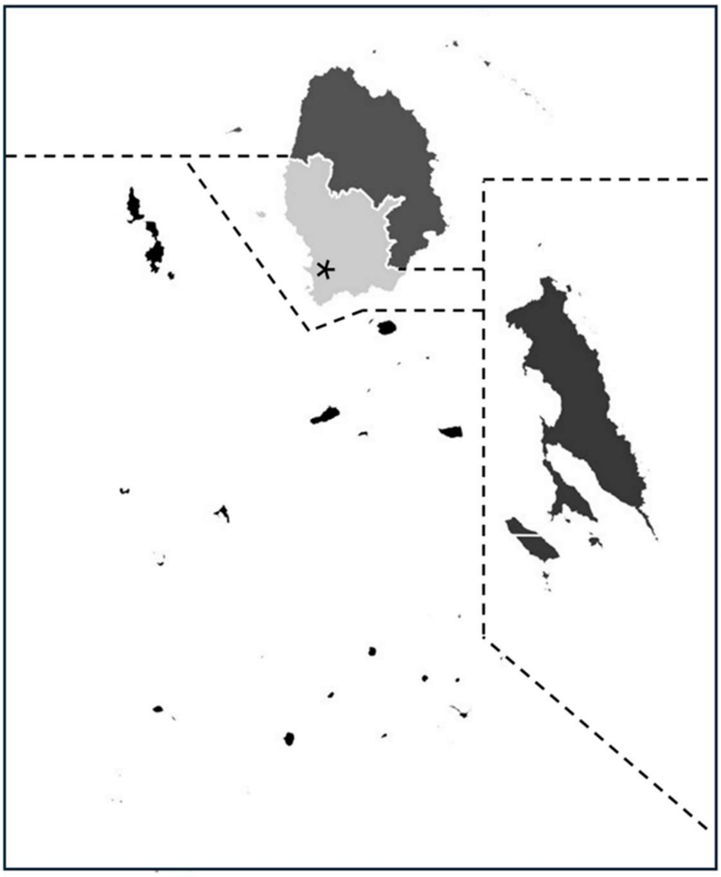

Northern Division

N = 14

S. Stercoralis IgG ELISA seropositivity = 42.86%

Province	N	IgG ELISA +ve	% IgG ELISA positive
Cakaudrove	10	5	50.00
Macuata	3	0	00.00
Bua	1	1	100.00

Eastern Division

N = 17

S. Stercoralis IgG ELISA seropositivity = 47.05%

Province	N	IgG ELISA +ve	% IgG ELISA positive
Kadavu	7	3	42.85
Lomaiviti	6	3	50.00
Lau	4	2	50.70

Supplementary figure 3. ROC curves for eosinophil count prediction of faecal positivity for *S. stercoralis*, hookworm, or both helminths combined. AUC = Area under the curve

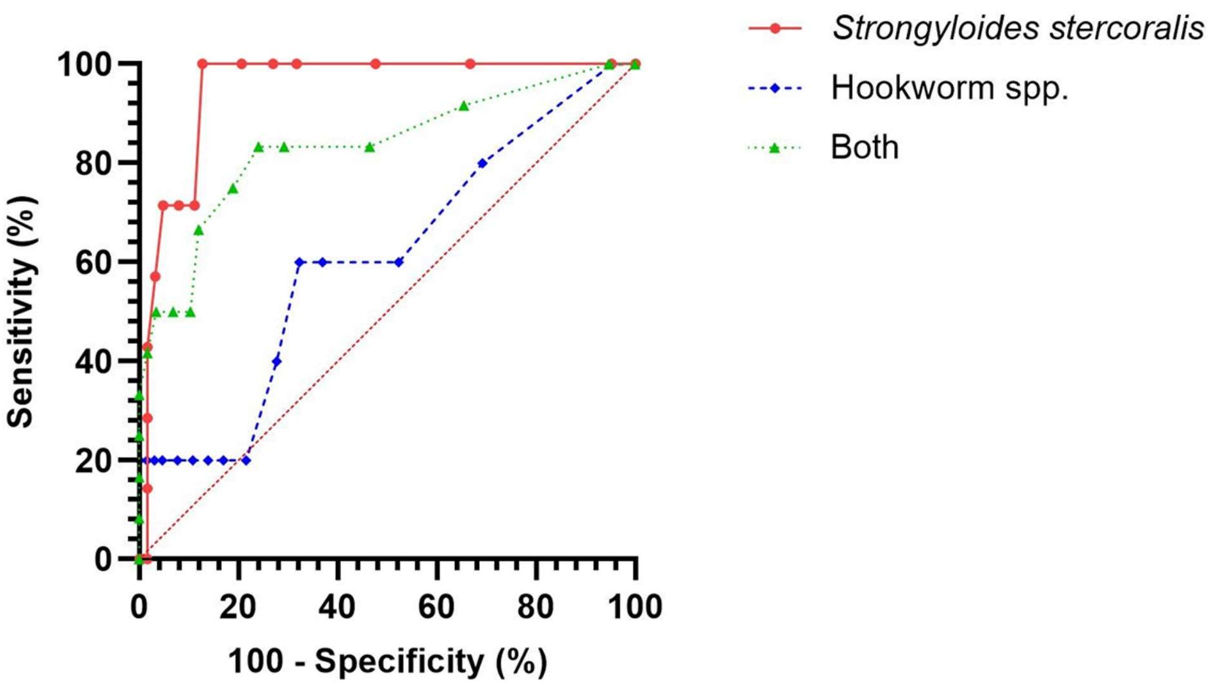

	AUC	95% CI	P value
S. stercoralis	0.950	0.897-1.000	0.0001
Hookworm	0.605	0.339 0.870	0.438
Both	0.834	0.688-0.980	0.0003

Supplementary table 1. Forward and reverse primers for PCR of extracted DNA from *Strongyloides stercoralis* pooled larvae. Primer sequences, expected length of sequence, and annealing temperature shown. Sequences were obtained using conventional PCR for 18S rRNA Highly Variable Regions I and IV.

Primer name	Sequence (5'-3')	Length (bp)	Annealing temp (^{o}C)	Reference
HVR I Forward	GCTCATTATAACAGCTATAGACTACACGGTA	434 approx.	60	1.
HVR I Reverse	CCACAACAATCATTTTATGCACTTGG			
HVR IV Forward	CGGGCCGGACACTATAAGG	255 approx.	63	
HVR IV Reverse	ATCTCTAAACAGGAACATAATGATCACTAC			

1. Barratt, J.L.N., et al., A global genotyping survey of Strongyloides stercoralis and Strongyloides fuelleborni using deep amplicon sequencing. PLoS Negl Trop Dis, 2019. 13(9): p. e0007609

Supplementary table 2. *Strongyloides stercoralis* IgG ELISA results. Table showing number and percentage of positive and negative *S. stercoralis* IgG ELISA results, and mean and median Optical Densities (OD) for each group. Negative results are divided into negatives (OD < 0.185) and high negatives (OD ≥ 185 - < 0.200). Positive results are divided into weakly positives (OD ≥ 0.200 - < 0.250), standard positives (OD ≥0.250 - <1.000) and strongly positives (OD ≥ 1.000). OD values were not normally distributed, with a Shapiro-Wilk test significance of < 0.001. Median eosinophil counts, number with eosinophilia and percentage with eosinophilia are shown for each group. Note, eosinophilia totals may not match the total number of serological tests performed as not all participants had a valid differential white cell count available.

	No. of participants	%	Mean OD	Median OD	Median eosinophil count (10^9/L)	No. with eosinophilia (≥ 0.5 x 10^9/L)	% with eosinophilia
**All negative**	**161/248**	**64.9**	**0.098**	**0.096**	**0.2**	**23/148**	**15.5**
Negative (OD < 0.185)	154	62.1	0.095	0.092	0.2	22/141	15.6
High Negative (OD ≥ 0.185 -< 0.200)	7	2.8	0.190	0.189	0.2	1/7	14.3
**All positive**	**87/248**	**35.1**	**0.721**	**0.417**	**0.5**	**45/81**	**55.6**
Weakly positive (OD ≥ 0.200 - < 0.250)	23	9.3	0.227	0.230	0.2	5/22	22.7
Standard positive (OD ≥ 0.250 - < 1.000)	40	16.1	0.489	0.396	0.5	21/36	58.3
Strongly positive (OD ≥ 1.000)	24	9.7	1.580	1.399	0.7	19/23	82.6
**Total**	**248**		**0.317**	**0.133**	**0.3**	**68/229**	**29.7**

Supplementary table 3. Diagnostic test results in 5 participants with positive/equivocal faecal real-time PCR for hookworm species. For definitions of semi-quantitative results for faecal PCR and serological results, see manuscript text.

Age (years)	Years since migration to UK	Eosinophil count (10^9/L)	FEA microscopy	Charcoal culture	Real-time PCR	*S. stercoralis* IgG ELISA OD value	*S. stercoralis* IgG ELISA result
31	16	0.1	-	+	+	0.352	++
21	20	4.0	-	-	+	0.527	++
20	1	0.5	-	-	+	0.146	-
43	3	0.2	-	-	equiv	0.492	++
29	1	0.6	-	-	equiv	0.230	+

Supplementary table 4. Values for sensitivity and specificity calculations for diagnostic tests for of *Strongyloides stercoralis*. Using a reference standard of A. Faecal qPCR positive/equivocal result or B. S. stercoralis IgG ELISA positive result. 95% confidence intervals shown in brackets.

A. Faecal qPCR as reference standard

	Charcoal culture		Microscopy		*S. stercoralis* IgG ELISA	
qPCR	Positive	Negative	Positive	Negative	Positive	Negative
Positive	2	5	1	6	7	0
Negative	0	67	0	67	27	38
Sensitivity (%)	28.6 (3.7 - 71.0)		14.3% (0.4 - 57.9)		100.0 (59.0 - 100.0)	
Specificity (%)	100.0 (94.6 - 100.0)		100.00 (94.64 - 100.0)		58.5 (45.6 - 70.6)	

B. *Strongyloides stercoralis* IgG ELISA as reference standard

	Charcoal culture		Microscopy		qPCR	
Serology	Positive	Negative	Positive	Negative	Positive	Negative
Positive	2	32	1	33	7	27
Negative	0	38	0	38	0	38
Sensitivity (%)	5.9 (0.7 - 19.7)		2.9 (0.1 - 15.3)		20.6 (8.7 - 37.9)	
Specificity (%)	100.0 (90.8 - 100.0)		100.0 (90.8 - 100.0)		100.0 (90.7 - 100.0)	

Supplementary table 5. Table of travel to other *Strongyloides stercoralis* endemic regions by study participants. Number and percentage of participants who travelled to each region is shown, as well as the most frequently travelled to country in each region.

S. stercoralis endemic region	Number of participants	% (N=244)	Most frequent country	Number	% (N=244)
Fiji only	84	34.4			
Other:	160	65.6			
Middle East	106	43.4	Iraq	83	34.0
Asia (excluding SE Asia)	109	44.7	Afghanistan	99	40.6
Southeast Asia	8	3.3	Brunei	4	1.6
Sub Saharan Africa	72	25.5	Kenya	59	24.2
North Africa	2	0.8	Egypt	2	0.8
South and Central America	26	10.7	Belize	26	10.7
Oceania (excluding Fiji)	16	6.6	Australia	16	6.6
Caribbean	3	1.2	Barbados	3	1.2
Southern Europe	69	27.3	Cyprus	50	20.5

Supplementary table 6. Table of reported predominant living environment (urban or rural) of study participants. Numbers are shown for faecal methodologies and *S stercoralis* IgG ELISA.

	Rural	Urban	Equal rural/urban
S. stercoralis faecal positive/equivocal	6	1	0
Hookworm faecal positive/equivocal	3	2	0
S. stercoralis IgG ELISA positive	47	31	8
S. stercoralis IgG ELISA negative	52	98	7
All participants	100	129	15

Supplementary table 7. Symptomatology of participants shown by number of participants and percentage. Values are shown for all participants and those with positive diagnostic/equivocal faecal tests for *S. stercoralis* and hookworm spp, and positive *S. stercoralis* IgG ELISA.

		Any symptoms		Gastrointestinal symptoms							
		Yes	No	Diarrhoea	Abdo. pain	Blood in stools	Any GI	Rashes	Cough / SOB	Unexplained weight loss	Subjective fevers
All participants	Number	94	150	21	20	24	52	42	33	4	30
	% of group	38.5	61.5	8.6	8.2	9.8	21.3	17.2	13.5	1.6	12.3
S. stercoralis faecal positive/equivocal	Number	7	0	1	2	0	3	4	1	1	3
	% of group	100.0	0.0	14.3	28.6	0.0	42.9	14.3	57.1	14.3	42.9
Hookworm spp. faecal positive/equivocal	Number	3	2	2	0	0	2	0	1	0	1
	% of group	60.0	40.0	40.0	0.0	0.0	40.0	0.0	20.0	0.0	20.0
S. stercoralis IgG ELISA positive (any)	Number	49	37	12	11	11	27	27	13	2	20
	% of group	57.0	43.0	14.0	12.8	12.8	31.4	31.4	15.1	2.3	23.3

Supplementary table 8. Logistic regression analyses of association between *S. stercoralis* IgG ELISA positivity, travel to an S. stercoralis endemic area and residential environment in Fiji. Odds ratios (OR) were calculated for each variable individually, including age, and as part of a multiple variable analyses (including age and the reporting of any symptoms). 95% CI shown in brackets.

	Individual variable, no other variables included OR (95% CI)		Individual variable, age of participants included		All variables (including age and reporting any symptoms)	
Variable	OR	P value	OR	P value	OR	P value
Age	1.02 (0.99-1.06)	0.15	[redacted]	[redacted]	1.01 (0.96-1.05)	0.80
Travel to other endemic area	1.74 (0.98-3.09)	0.06	1.55 (0.78-3.08)	0.21	1.26 (0.60-2.66)	0.54
**Living in rural environment**	**2.43 (1.42-4.17)**	**0.001**	**2.40 (1.40-4.13)**	**0.002**	**2.04 (1.11-3.74)**	**0.02**
Living in medium HDI province	2.11 (1.09-4.09)	0.03	1.99 (1.02-3.89)	0.04	1.33 (0.62-2.86)	0.46

Table 9. Logistic regression analyses of possible associations between *S. stercoralis* IgG ELISA positivity and symptoms. Odds ratios (OR) were calculated for each variable individually, including age, and as part of a multiple variable analyses. Odds ratios and p values are shown, with 95% confidence intervals (CI) in brackets.

	Individual variable, no other variables included		Individual variable, age of participants included		All variables included	
Variable	OR<br>95% CI	P value	OR	P value	OR	P value
Age	1.02 (0.99-1.06)	0.15			1.02 (0.98-1.05)	0.42
Any symptoms	3.30 (1.90-5.71)	<0.001	3.17 (1.82-5.52)	<0.001	2.43 (0.87-6.78)	0.09
GI symptoms (any)	2.42 (1.29-4.51)	0.006	2.27 (1.20-4.28)	0.01	1.06 (0.42-2.69)	0.90
**Rashes**	**5.07 (2.45-10.50)**	**<0.001**	**4.88 (2.35-10.15)**	**<0.001**	**3.44 (1.43-8.31)**	**0.006**
Cough/shortness of breath	1.22 (0.57-2.59)	0.61	1.17 (0.55-2.50)	0.69	0.42 (0.16-1.12)	0.08
Subjective fevers	1.96 (0.995-3.87)	0.052	1.93 (0.98-3.83)	0.06	1.05 (0.42-2.65)	0.92
Weight loss	1.85 (0.26-13.33)	0.54	1.86 (0.25-13.62)	0.54	2.24 (0.27-18.59)	0.46

Participant Questionnaire

Unique Identifier Number: ________________

Please only answer questions which you would like to. Answers will remain anonymous. Not answering a question will not have any impact on your training/career or involvement in the study. Please ask an investigator if you need help with a question.

Date of joining the Armed Forces:___/____/______

Date of arrival in the UK: ___ /___ /______

Country of birth: ______________ Town of birth: ______________

Apart from the UK and your country of birth, have you travelled to/been deployed to any other countries? Yes/No

If yes, please list these with dates:
(More paper can be provided if needed)

Country:	Date:
______________________________	______
______________________________	______
______________________________	______
______________________________	______

Before moving to the UK, what was the name of the main City/Town/Village/Area you lived in? ______________________________

Did you live in an urban (town) or a rural (countryside) place, or both equally ______________________________

As far as you know, have you ever been treated for any of the following infections:

		Date/age (approximately):
Tuberculosis	Yes/ No / Don’t Know	______
Gut worms	Yes/ No / Don’t Know	______
Schistosomiasis (Bilharzia)	Yes/ No / Don’t Know	______
Skin infections/boils	Yes/ No / Don’t Know	______

Do you have, or have you experienced any of the following medical problems on a persistent (ongoing) basis? A member of the study team may contact you with follow up questions about the symptoms.

		Please give any details below:
Repeated or long-term diarrhoea/loose stools	Yes / No	
Blood in stools	Yes / No	
Abdominal pains	Yes / No	
Blood in urine	Yes / No	
Unexplained weight loss	Yes / No	
Coughing/shortness of breath/difficulty breathing	Yes / No	
Fevers	Yes / No	
Sweating at night	Yes / No	
Rashes	Yes / No	
Skin infections/boils	Yes / No	
Any other symptoms?	Yes / No	

Thank you for agreeing to take part in the study. If you have any questions, please contact:

Dr William Nevin
